# S04-4: The HEPA PAT: An in-depth tool to audit HEPA policies at national level

**DOI:** 10.1093/eurpub/ckae114.214

**Published:** 2024-09-26

**Authors:** Sonja Kahlmeier

**Affiliations:** Swiss Distance University of Applied Science FFHS

## Abstract

**Purpose:**

The Health-Enhancing Physical Activity Policy Audit Tool (HEPA PAT) a standardized protocol for the detailed compilation and communication of country-level policy responses on physical inactivity. This presentation aims to briefly present the structure of the tool as well as its use in PA policy research.

**Methods:**

The 29 open-ended and closed questions of the HEPA PAT are structured around a set of 17 key criteria, which were identified based on experiences from several previous guidelines and comparisons of physical activity policy. It supports standardized data collection and is to be completed collaboratively by a team of national stakeholders from all relevant political sectors. However, the process of its application differed across countries.

**Results:**

The HEPA PAT has been applied in 13 European countries (Belgium, Croatia, Finland, France, Germany, Ireland, Italy, the Netherlands, Norway, Poland, Portugal, Slovenia, Switzerland). These studies provided a detailed overview of physical activity policies for the different sections of the tool such as, for instance, leadership and partnerships, policy documents, evaluation, and funding. A recent study has shown that the tool can be applied to understand aspects influencing the policy process (agenda-setting, policy formulation, decision-making, policy implementation, evaluation).

**Conclusions:**

The studies based on the HEPA PAT showed in which areas governments are already active to promote physical activity and what improvements can be made. Cross-country comparisons also showed that procedures and timelines of using the HEPA PAT have to be adapted to national contexts. Overall, the instrument can make an important contribution to understanding and informing physical activity policy, especially when used as an add‐on to regular monitoring tools like the EU HEPA Monitoring Framework. The PAT completion also fostered new connections with policy makers and officials in government departments and among HEPA stakeholders within and outside of government, which in itself was seen to be extremely valuable.

**Funding Source:**

No funding

